# Supplementary material for: Patterns of Intron Gain and Loss in Fungi
Source: PLoS Biol. 2004 Nov 30;2(12):e422. doi: 10.1371/journal.pbio.0020422 (PMC532390; doi:10.1371/journal.pbio.0020422)
Supplement: Table S1 — Also available at http://genes.mit.edu/NielsenEtAl/. (4.3 MB ZIP). [file pbio.0020422.st001.zip › NielsenEtAl/html/817.html]

AN3466.1.NCU02438.1.MG03149.1.FG07970.1


```
 CLUSTAL W (1.82) Multiple Sequence Alignments - Introns Inserted


Sequence 1: MG03149.1	421 aa
Sequence 2: FG07970.1	421 aa
Sequence 3: NCU02438.1	423 aa
Sequence 4: AN3466.1	453 aa
Alignment Length: 453 aa
Number Identitical Residues: 266 aa
Alignment Score (without introns) 10958


MG03149.1 	MFSRR-----------~----------LITAARLVP----VSQAT~SQPLRRFPLHRHFQ
NCU02438.1	MLYKG-----------~----------LRLAARAAP----KLALC~NATPALRQLPLQFQ
FG07970.1 	MLSRS-----------~----------IATASRMVP----ARVLR~PRAHPLVMLPAMMQ
AN3466.1  	MASRLSLRQFSAQRLR2TFTTSKGSENARSASGLIQGLQKGPALR2NGFSRVNVIPISNY
          	*  : :  . ::.    : :::..:..   *:    . ..              :     

MG03149.1 	HVRTYA~DQVVKVPQMAESISEGTLKQFTKQ~VGDFVEQDEELATIETDK0IDVAVNAPA
NCU02438.1	HVRTYA~DQVIKVPQMAESISEGTLKQWNKK~VGDYVEQDEEIATIETDK0IDVAVNAPE
FG07970.1 	TVRTYA~DSVIKVPQMAESISEGTLKQFSKS~IGDYVAQDEEIATIETDK0IDVAVNATE
AN3466.1  	QVRTYA1DTVVKVPQMAESITEGTLKQFSKQ1VGDYVERDEEIATIETDK0IDVSVNAPE
          	 ***** * *:*********:******:.*. :**:* :***:******* ***:***. 

MG03149.1 	AGTIKELLASEEDTVVVGQDLIRLELGGAP-AEGAEKKSE--KPQETQAEKKPEPKEESK
NCU02438.1	AGTIKEFLVNEEDTVTVGQDIVRLELGGAPKEGGAEKPAA--SESKEAAPKDSAPAPEKA
FG07970.1 	AGTIKEFLVAEEDTVTVGQDLVRIELGGEPSGDKKEAPKE--EPKKSESESKPEPKQESA
AN3466.1  	SGVIKELLVNEEDTVTVGQDLVKLEAGGTPEKKSEEATEKPKEPASTGSEAEKPKEPESA
          	:*.***:*. *****.****::::* ** *.    *    ...  .  :  .     *. 

MG03149.1 	PEPTKKEEPAPSKKQESAPQPE-KKETKQPAK--ESSPATLGNREERR0VKMNRMRLRTA
NCU02438.1	PEP-KKETKPAAAPAPTPAKKE-TPAPKQESTPAKEAPAALGNREERR0VKMNRMRLRIA
FG07970.1 	PEP-KKEPAAASSKPEAPRQPE-KKESKSESS--ASSGSSMGNREERR0VKMNRMRLRIA
AN3466.1  	PSSSAPEKSTSSTKAPQAETSKPTQEVASKSRPTEEAKPALGNREERR0VKMNRMRLRIA
          	*..:  *  ..:     .   :..    . : .: .: .::******* ********* *

MG03149.1 	ERLKQSQNTAASLTTFNEVDMSSLMEFRKLYKDDVMKKTGVKLGFMSAFSRAAVLAMRDI
NCU02438.1	ERLKQSQNTAASLTTFNEVDMSGLMDFRKQYKDEILKKTGVKLGFMSAFSRACVLAMRDI
FG07970.1 	ERLKQSQNTAASLTTFNEVDMSNIMEFRKLYKEDVLKKTGVKLGFMSAFSRACVLAMRDL
AN3466.1  	ERLKQSQNTAASLTTFNEVDMSSLMEFRKLYKDEILKKTGVKLGFMSAFSRACVLAMKDV
          	**********************.:*:*** **::::****************.****:*:

MG03149.1 	PAVNASIEGPNGGDTIVYRDYVDISLAVATEKGLVVPVVRNTESMDMITIEKSIAEMGAK
NCU02438.1	PAVNASIEGPNGGDTIVYRDYVDISVAVATEKGLVTPVVRNVEAMDLVGIEKSIADMGKK
FG07970.1 	PAVNASIEGPNGGDTIVYRDYVDISVAVATEKGLVTPVVRNVESMDMIGIEESIADMGKK
AN3466.1  	PAVNASIEGPNGGDTIVYRDYVDISVAVATEKGLVTPVVRNAETMDLVGIEKSIADLGKK
          	*************************:*********.*****.*:**:: **:***::* *

MG03149.1 	0ARAGKLTIEDMAGGTFTISN~GGVFGSLMGTPIINLPQTA~VLGLHAIKDRPVVVNGKI
NCU02438.1	0ARDGKLTIEDMAGGTFTISN~GGVFGSLMGTPIINLPQSA~VLGLHAIKERPVAVNGKV
FG07970.1 	0ARDNKLTIEDMAGGTFTISN~GGVFGSLMGTPIINLPQSA~VLGLHAIKERPVAVNGKI
AN3466.1  	0ARDNKLTIEDMAGGTFTISN0GGVFGSLMGTPIINLPQTA1VLGLHAIKDKPVAIGGKV
          	 ** .**************** *****************:* ********::**.:.**:

MG03149.1 	EIRP~MMYLALTYDHRLLDGREAVSFLVK~IKEFIEDPRRMLL-
NCU02438.1	EIRP~MMYLALTYDHRLLDGREAVQFLVK~VKEYIEDPRKMLL-
FG07970.1 	EIRP~MMYLALTYDHRLLDGREAVQFLVK~VKEYIEDPRRMLL-
AN3466.1  	EIRP0MMYLALTYDHRLLDGREAVTFLVK0VKEYIEDPRRMLLG
          	**** ******************* **** :**:*****:***.
```
